# Working principle and relevant physical properties of the Swiss Liquid Jet Aesthesiometer for Corneal Sensitivity (SLACS) evaluation

**DOI:** 10.1111/opo.12962

**Published:** 2022-02-14

**Authors:** Daniela S Nosch, Matthias Oscity, Peter Steigmeier, Emanuele Käser, Markus Loepfe, Roland E Joos

**Affiliations:** ^1^ Institute of Optometry University of Applied Sciences and Arts Northwestern Switzerland (FHNW) Olten Switzerland; ^2^ Institute of Sensors and Electronics University of Applied Sciences and Arts Northwestern Switzerland (FHNW) Windisch Switzerland

**Keywords:** cornea, corneal sensitivity, liquid jet aesthesiometry

## Abstract

**Purpose:**

To describe and evaluate relevant physical properties of the Swiss Liquid Jet Aesthesiometer for Corneal Sensitivity (SLACS) for ocular surface sensitivity measurement.

**Methods:**

Characteristics of Liquid Jet (LJ) droplets (consisting of isotonic saline solution) were analysed: vertical and horizontal displacement and speed of LJ droplets were recorded with the aid of the High Speed Photron FASTCAM NOVA S6 camera (stimulus duration: 40 ms). Stimulus mass was assessed for 20 sets of 10 LJs with aid of a microbalance (pressure range of 100–1500 mbar).

**Results:**

Because continuous flow LJ disintegrated into droplets in the lower pressure range (<700 mbar), pulsed stimuli were applied in order to obtain similar stimulus characteristics across the applied pressure range. For all measurements, very little variability was observed. Vertical and horizontal displacement did not exceed 0.13 mm in either direction. The mass per shot showed an unexpected cubic dependency on pressure. Up to approximately 700 mbar, LJ speed showed an almost linear relationship. For the pressure range of >700–1500 mbar, variability increased and speed decreased compared to the expected in a linear manner. However, this may be caused by the difficulty of identifying pattern changes of LJ droplets from one high speed image frame to the next with increasing stimulus speed, when determining LJ speed via pixel count.

**Conclusions:**

Swiss Liquid Jet Aesthesiometer for Corneal Sensitivity was shown to deliver fine droplets with a pulsed stimulus mode, in a repeatable manner with precise localisation to the ocular surface. Very little variability was observed in LJ speed and mass for the typical pressure range required for clinical sensitivity measurements.

**Key points**

- This article presents relevant physical properties of the new Swiss Liquid Jet Aesthesiometer for measurement of corneal sensitivity.
- Corneal sensitivity may be affected in dry eye disease, corneal dystrophies, refractive surgery, corneal transplants, contact lens wear or with systemic associations causing peripheral neuropathy (e.g., diabetes).
- The new Swiss Liquid Jet Aesthesiometer is shown to deliver fine droplets with a pulsed stimulus mode, in a repeatable manner with precise localisation to the ocular surface.

## INTRODUCTION

The human cornea is innervated with a dense network of sensory nerves that respond to mechanical, chemical and thermal stimulation.^1^, ^2^, ^3^, ^4^ These sensory nerves serve four functions: detection of foreign bodies or noxious substances, detection of tear film thinning to promote tear production, detection of changes in the tear film to promote blinking and a neurotrophic role in the maintenance of the corneal epithelium.^1^, ^5^, ^6^, ^7^, ^8^, ^9^

From a clinical and research perspective, it is of great interest to quantify ocular surface sensation in eyes with altered nerve functions in the sub basal nerve plexus such as in dry eye disease,^10^, ^11^, ^12^, ^13^, ^14^ corneal dystrophies (e.g., keratoconus),^15^ before and/or after refractive surgery,^16^, ^17^, ^18^ in corneal transplants,^19^, ^20^ with contact lens wear^21^ or with systemic associations causing peripheral neuropathy (e.g., diabetes).^22^

Currently, the tactile Cochet Bonnet aesthesiometer (Luneau Technology, luneautech.com) is the only commercially available instrument to test corneal sensitivity. It employs a fine nylon filament (0.12 mm diameter) that is applied to the cornea with varying pressures by adjusting its length to produce different stimulus intensities to the ocular surface (max. length 6 cm).^23^ However, is rarely used in clinical practice due to limitations such as risk of abrasion of the epithelial surface; questionable reliability; alignment and precision difficulties; limited stimulus range and resolution and the influence of ambient humidity on how the nylon filament bends.^24^, ^25^, ^26^, ^27^ Various prototypes of non‐contact air jet aesthesiometers were developed to overcome most of these problems.^28^, ^29^, ^30^, ^31^, ^32^ The airflow affecting the cornea is supposed to produce a mechanical stimulus by deforming the cornea when its temperature matches the ocular surface temperature (OST). It may also act as a heating or cooling stimulus, when it is warmed or kept at room temperature. A chemical stimulus can be also generated using CO_2_ gas.^28^, ^29^ A cooling stimulus will excite the cold temperature sensitive nerve endings, and a mechanical stimulus should be sensed by the mechano‐nociceptors and polymodal nociceptors when sufficient degree of corneal deformation can be produced by the air jet stimulus. For a true mechanical stimulus, it is important to eliminate any thermal components, which is difficult since the air jet will cause a flow rate‐dependent evaporative cooling effect on the wet cornea.^33^ It is also problematic that upon arrival at the ocular surface, the air jet stimulus disperses in a lateral motion over the entire corneal surface, thereby creating a stimulus footprint that is hard to determine.^34^

In order to overcome these deficiencies, a novel, non‐invasive liquid jet prototype employing small droplets of isotonic saline solution was presented by Ehrmann et al.^35^ The liquid jet stimulus exits a microvalve (with 0.1 mm diameter) mounted on a slit lamp and equipped with a heating coil and a temperature sensor, in order to match the ocular surface temperature for the generation of a mechanical stimulus. Stimulus strength is controlled by switching the microvalve on and off at a high frequency up to 4 kHz, with a minimum ‘on’ period of 0.15 ms. The pressure setting is fixed at 300 mbar, and stimulus intensity increases with the duration that the microvalve is opened from a minimum of 0.15 ms to a typical maximum of 100 ms per stimulus, required to elicit corneal sensation, i.e., it is quantified as the total volume or corresponding mass ejected for each stimulus: the pulse ratio (ratio of open / closed mode of the exit valve). However, it is unclear how this change of pulse ratio and the resulting change in liquid volume may alter the strength of the stimulus, as a pressure difference through the exit nozzle should be required, either by a pressure difference or variable nozzle diameter. If a change in these characteristics (stimulus duration/pulse ratio) affects the physiological response of the corneal nerves, then this requires neurological, rather than a physical explanation.

The authors of this article developed a new, modified liquid jet aesthesiometer, the Swiss Liquid Jet Aesthesiometer for Corneal Sensitivity (SLACS), whereby stimulus intensity was controlled with variable pressure levels, with a fixed stimulus duration of 40 ms. The aim of this study was to describe relevant physical properties of this new modified liquid jet aesthesiometer, and to validate it for clinical ocular surface sensitivity measurement.

## METHODS

### Instrument description

**FIGURE 1:**
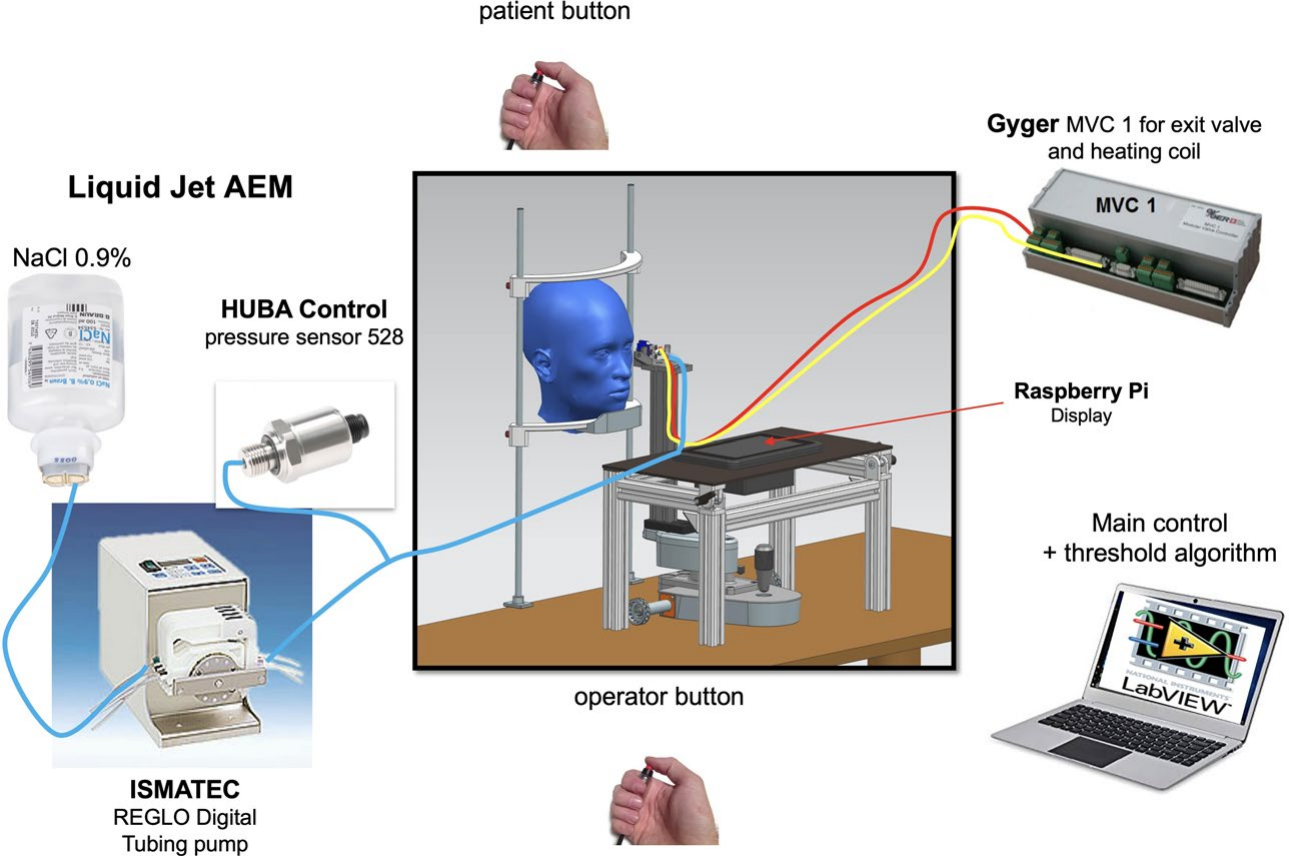
Diagram of the Swiss Liquid Jet Aesthesiometer for Corneal Sensitivity (SLACS)

**FIGURE 2:**
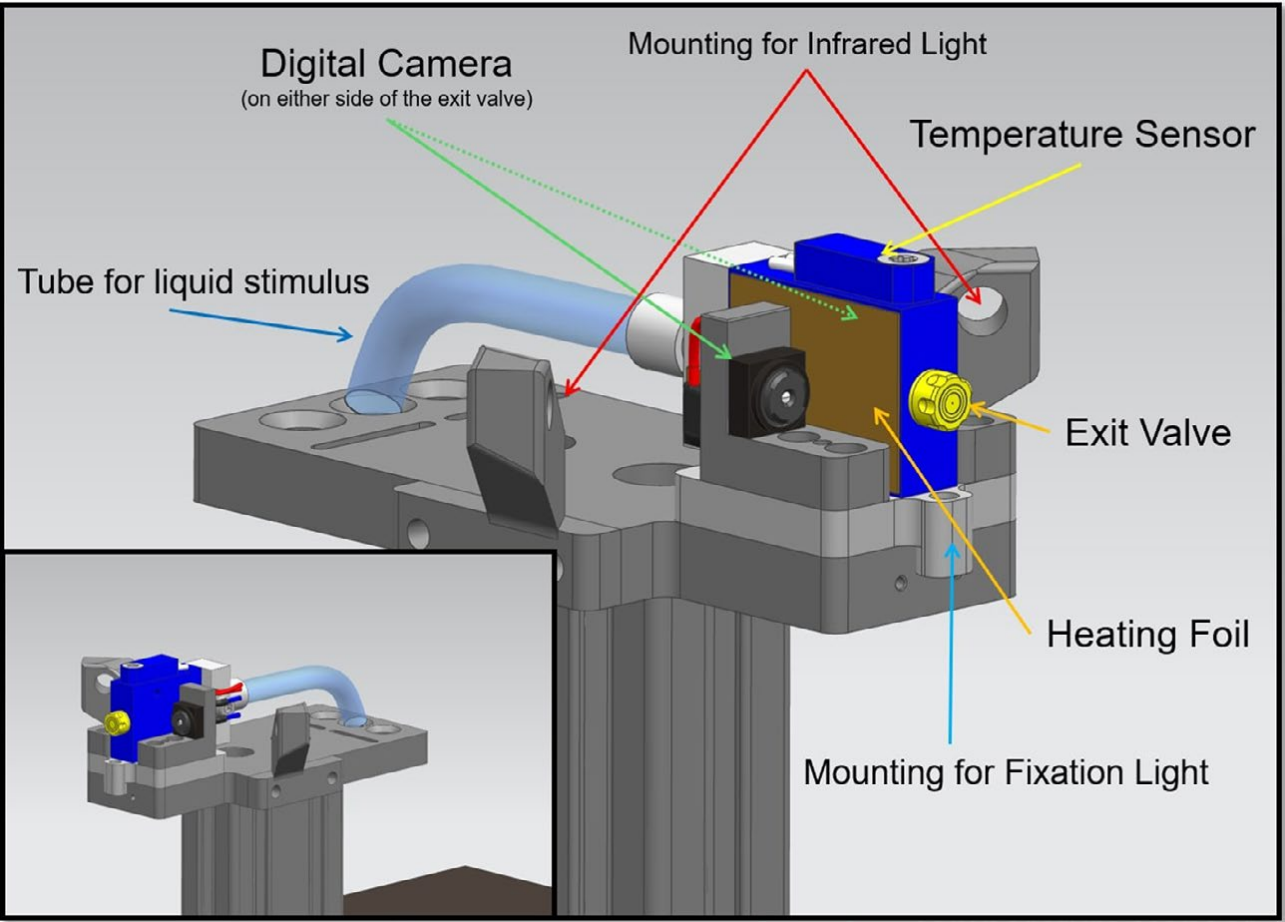
Close‐up diagram of the Swiss Liquid Jet Aesthesiometer for Corneal Sensitivity (SLACS)

**FIGURE 3:**
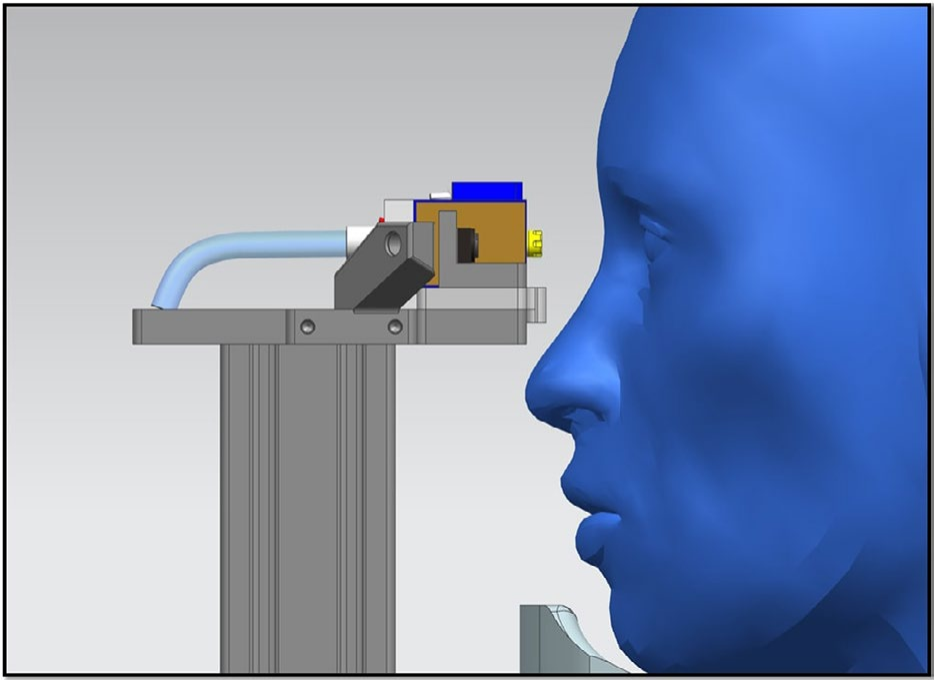
Lateral view of Swiss Liquid Jet Aesthesiometer for Corneal Sensitivity (SLACS) in front of the subject's eye

The prototype instrument comprises a REGLO Digital tubing pump (Ismatec, ismatec.com); a pressure sensor 528 (Huba Control, hubacontrol.com); a microvalve with a diameter of 0.1 mm (SMLD 300G H J0.1 T1 M F M6x0.75, Gyger, fgyger.ch) equipped with a heating coil, temperature sensor and a modular microvalve regulator (MVC 1.0 AH, Gyger, fgyger.ch); Raspberry Pi camera with display for fixation control on the central cornea and infrared light; a PC with LabView main control and an integrated threshold software algorithm for determination of ocular surface sensation threshold, as well as a pushbutton for both (Figures 1, 2, 3).

**FIGURE 4:**
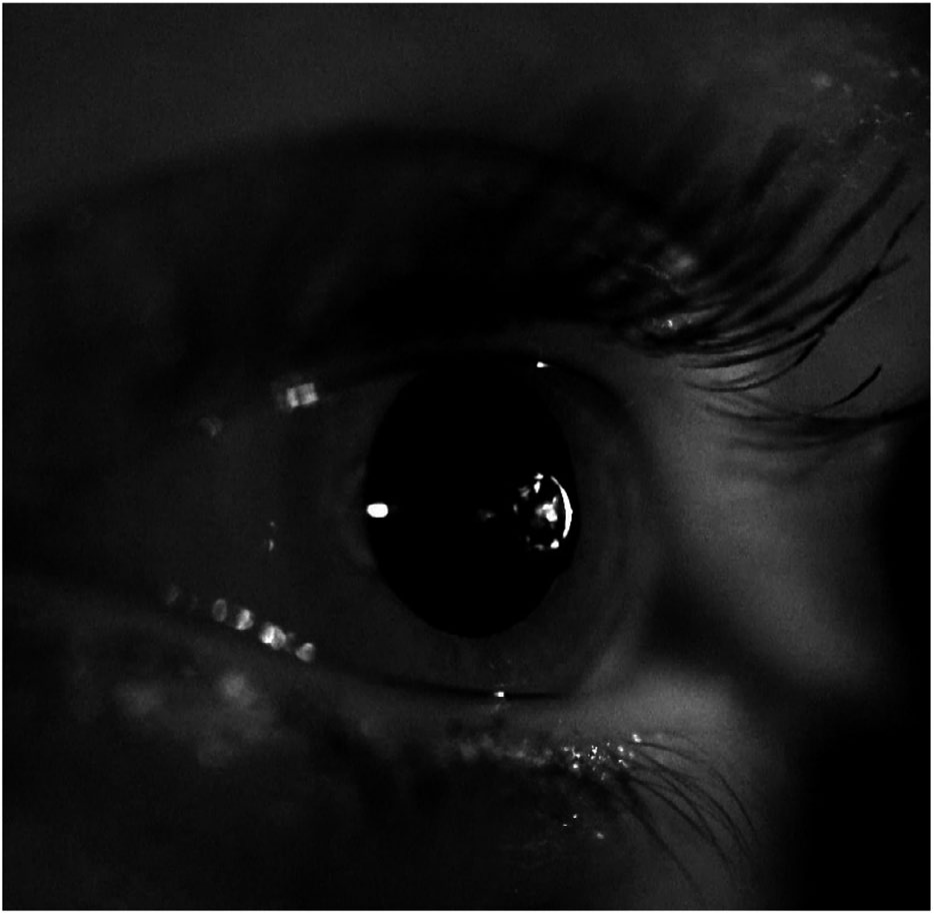
Liquid jet arriving at the corneal surface

The subject's head is positioned on a chin rest. A liquid jet (balanced salt solution 0.9% with a pH value and osmolarity similar to the tear film) at a temperature to match the ocular surface temperature is applied to the ocular surface with low pressure (typically up to 1000 mbar) and low volume (3.29 μL at 1000 mbar and valve opening time of 40 ms). The distance between the ocular surface and the exit valve should be sufficient to allow free blinking, but not too large as this may cause dispersion or deviation of the liquid jet. Hence, a distance of 15 mm was considered to be ideal. Figure 4 illustrates the liquid jet arriving at the corneal surface.

For determination of the threshold for mechanical stimulation, the pressure of the droplets during a stimulus duration of 40 ms is varied. A threshold for thermal stimulation can be determined by warming or cooling the stimulus above or below the ocular surface temperature, at a sub‐threshold level for mechanical stimulation. Alternatively, the pH of the stimulus may be increased or decreased to create a chemical stimulus.

A test procedure for determination of corneal sensitivity involves a software algorithm that randomly presents liquid jet stimuli above or below threshold, without any examiner's input on the choice of stimulus intensities presented. The subject provides ‘felt’ or ‘not felt’ feedback via a handheld pushbutton, thus avoiding direct interaction between the subject and the examiner. In this way, the threshold determination is believed to be independent of the examiner's influence. The final number of presented stimuli depends on the threshold calculation of the software algorithm, and is influenced by the reliability of the subject's responses.

To estimate the accuracy and efficiency of the algorithm, several simulations with various subject‐models were performed to minimise the number of presented stimuli as well as the threshold estimation error. This algorithm narrows the intensity limits throughout the procedure, and generates random intensities within the applied intensity range. Stimuli were presented in groups of two ‐ one from the upper half and one from the lower half of the current intensity range ‐ whereby the order of presentation as well as the intensity within each half of the intensity range were determined randomly using a uniform distribution for each half.

**FIGURE 5:**
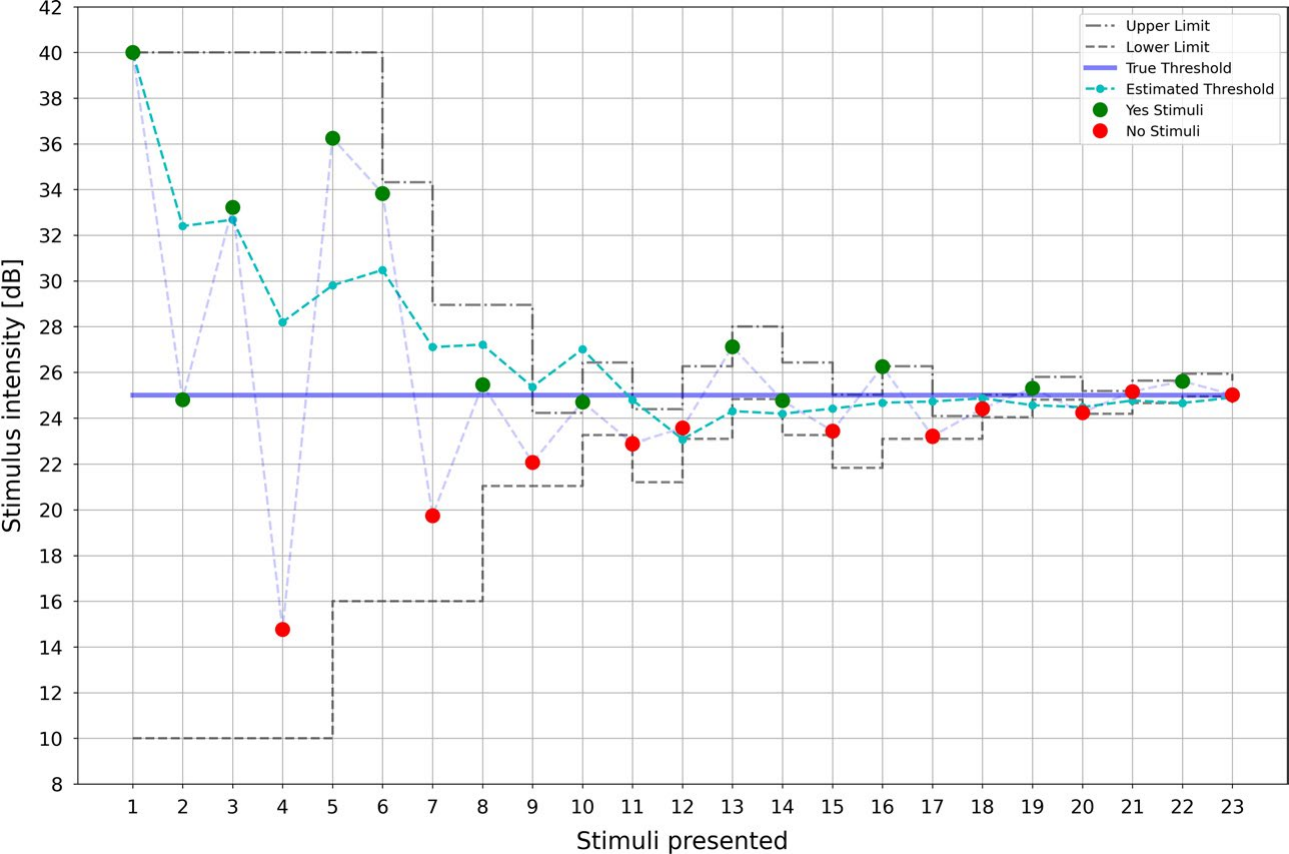
Example for determination of a threshold with stimulus intensity [dB] on the y‐axis and the number of stimuli presented to the ocular surface on the x‐axis. Green dots represent ‘yes’ responses and red dots represent ‘no’ answers. The dotted grey lines represent the upper and lower stimulus range limits, while the dotted turquoise line represents estimated thresholds and the blue line the true threshold

Changes in stimulus intensity range decreased in a linear manner to a minimum change of 0.6 dB, i.e., until an intensity range of 0.6 dB is reached – this represents the minimum intensity range. The aim was to arrive at a stimulus range no larger than 0.6 dB after a maximum of 16 to 20 stimulus presentations. The following conditions were applied for threshold determination after each stimulus: a minimum number of six stimuli have to be presented and only the last six stimuli contribute to the threshold calculation. The last six stimuli must have a standard deviation of ≤0.8 dB (Figure 5).

This algorithm was tested with the use of subject models. An inverse logit function $R\left(I\right)=\frac{1}{1+e^{‐k*\left(I‐I_{0}\right)}}$ was applied to model the subjects’ responses, where $I_{0}$ is the threshold and k is the steepness of the function. The parameter k may be interpreted as the reliability of a subject to answer correctly to a given stimulus (assessing if ‘no’ answers are below threshold and ‘yes’ answers are above threshold) and R(I) as the probability to answer with a ‘yes’ at the given intensity I. For example, at $I=I_{0}$, the probability to answer with ‘yes’ is 50%.

Various simulation series were performed to evaluate the efficiency and accuracy of the algorithm:

- Threshold $I_{0}$ and steepness k remained constant, while the desired accuracy (tolerance) was changed in 0.1 dB steps starting at 0.4 dB and ending at 0.9 dB;
- The desired accuracy and threshold $I_{0}$ remained constantwhile the steepness k was changed;
- Tolerance and steepness remained constant, while the threshold $I_{0}$ was changed;
- A constant number of stimuli was presented to a subjectmodel with constant threshold and steepness, while ignoring the condition whether the threshold was found or not.
- The first three types of simulations were truncated to a maximum of 50 stimuli per subject, should the algorithm not converge. For each simulation type the number of stimuli presented, the standard deviation and the real deviation from the true threshold were calculated.

It was concluded that the algorithm performs well and has no significant bias.

### Procedure for validation of the working principle

Ambient temperature was kept at 22.5 ± 1.2°C and humidity was 45% ± 2%. The valve opening time was set at 40 ms.

#### Liquid Jet analysis with aid of a high speed camera

The Liquid Jet was analysed with aid of a high speed camera (FASTCAM NOVA S6, Photron, photron.com); resolution: 1024 × 1024 pixels, operated at 512 × 96 pixels, 1 pixel ~35.81 µm ~36 µm) across a typical pressure range for corneal sensitivity measurement (200–1000 mbar, according to unpublished clinical data). Continuous liquid jets with a duration of 40 ms and a temperature of 36°C were recorded at pressures of 200, 250, 350, 500 and 1000 mbar. Because the liquid jet disintegrated into droplets in the lower pressure range before it arrived at the ocular surface at a distance of 15 mm from the exit valve (see Results section), in contrast with LJ at higher pressures that did not disintegrate, pulsed liquid jets with the same duration of 40 ms and a temperature of 36°C were additionally recorded, whereby each pulsed jet consisted of 40 intervals, with a valve opening time of 950 µs per each interval cycle of 1000 µs. For all subsequent validation measurements in this study, this pulsed type of stimulus was applied. In addition, the arrival of the pulsed Liquid Jet was recorded from a semi‐lateral view.

#### Stimulus mass determination

Ten liquid jets over a pressure range of 100–1500 mbar with a temperature of 36°C were sent at a cone onto a double sheet of blotting paper that was placed with its open side in front of the exit valve, at a distance of 15 mm. The difference of weight of the blotting paper was subsequently recorded with aid of a microbalance (model 15907500, Fisherbrand, fishersci.co.uk), resolution 0.1 mg. The cone shape of the blotting paper for liquid collection allowed recollection of repelling drops, hence minimising evaporation and rejection losses that may reduce subsequent weight measurement. Each set of 10 liquid jets (40 ms duration for each liquid jet with intervals of 2 s between each shot = 18.8 s per pressure measurement) was carried out four times and repeated on four other dates, resulting in 20 measurements per pressure.

#### Vertical and horizontal displacement of the Liquid Jet

Four liquid jets of each pressure setting (same characteristics as above) were recorded with the aid of the same Photron FASTCAM NOVA S6 high speed camera at a perpendicular angle from both the side and above the stimulus.

#### Liquid Jet speed

**FIGURE 6:**
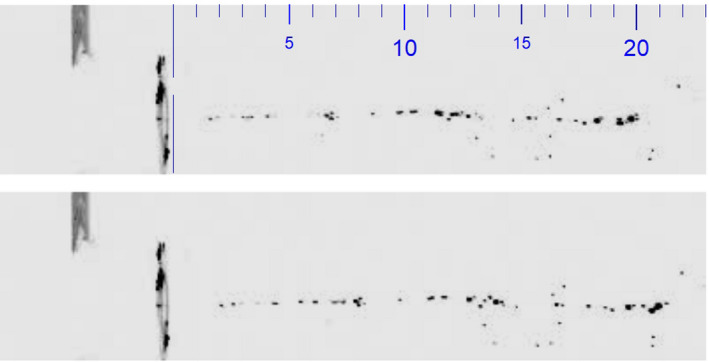
Jet images with a time difference of ten time frames: first frame in the top picture and ten frames later in the bottom picture; the patterns are similar but not identical. There is a very small shift of the overall pattern towards the right, as the droplets travel from left to right. The scale is marked in mm

Liquid Jet speed was analysed with aid of the same high speed camera, equipped with 8 GByte RAM and allowing for full size image (1024 * 1024 pixels at 12 bit depth) rates of 6400 frames per second for roughly 1.0 s. In order to investigate the nature of the beam while traveling a short distance of about 15–35 mm, only a region of interest of the image sensor was used: namely, 1024 pixel wide and 96 pixels high. By restricting the region of interest, the frame rate could be set to 50,000 frames per second. With use of high intensity LED illumination, the f‐number was set to 1/32, thus allowing for a maximum depth‐of‐field. Magnification was roughly 2:1. The reduced depth‐of‐field was critical. When considering the pattern of the Liquid Jet at various times, it changed continuously, with some droplets fading off while others seemed to grow and appear with higher intensity. In addition, there was an overall travel to the right due to the speed of the jet. In order to estimate the average speed of the Liquid Jet, the number of pixels was determined, until a best match between the two patterns was obtained. An Image Shift Algorithm was developed that was deemed to deliver sufficient reliability and precision in the context of this study (R software version 4.0.2, The R Project for Statistical Computing, r‐project.org) (Figure 6).

## RESULTS

### Liquid Jet analysis with aid of a high speed camera

**FIGURE 7:**
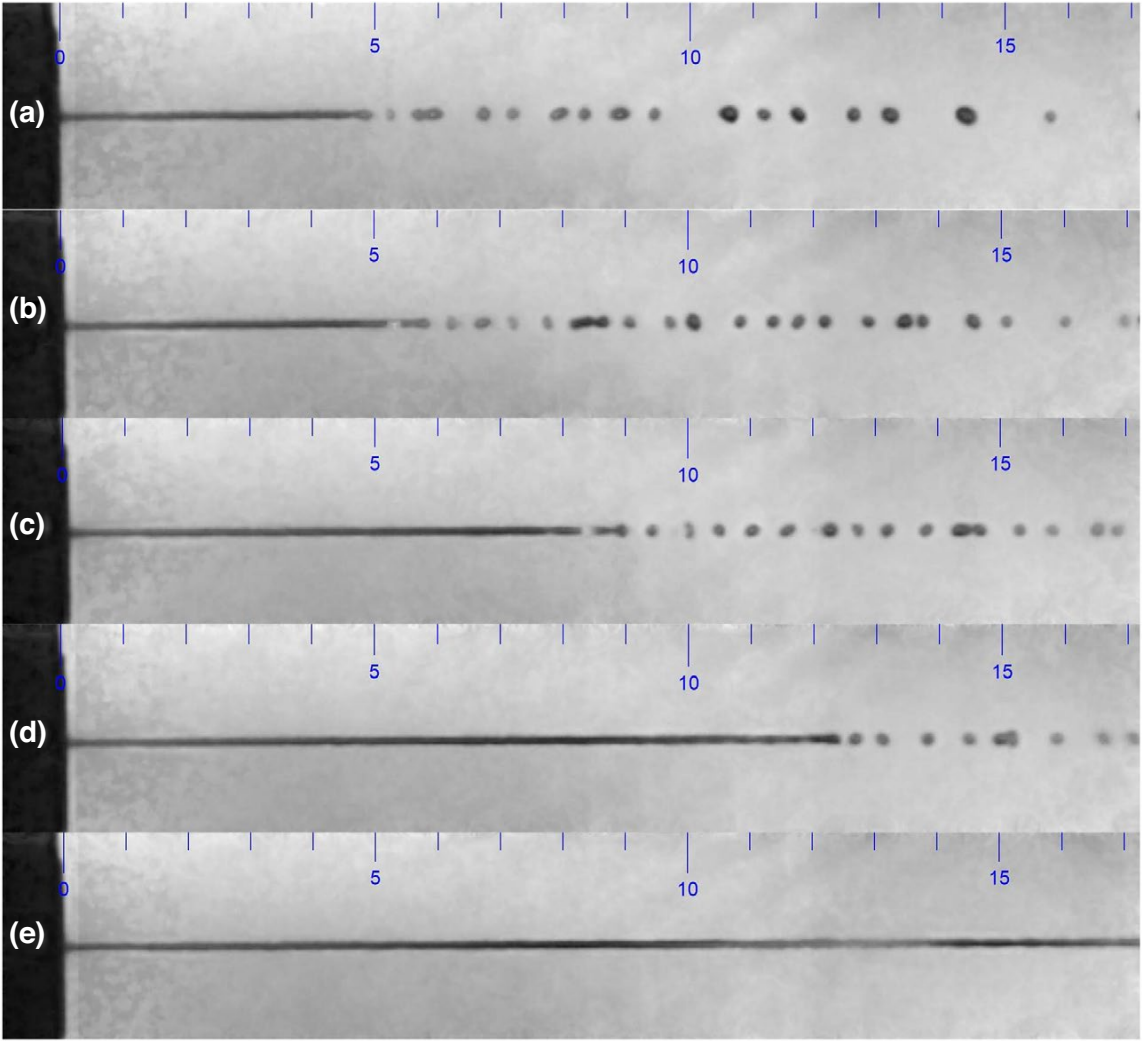
Continuous Liquid Jet at pressures of (a) 200, (b) 250, (c) 350 and (d) 500 mbar (top four figures, from top to down), disintegrating into droplets at a distance of 5 mm from the exit valve. No disintegration of the continuous Liquid Jet at a pressure of 1000 mbar was seen in the bottom figure (e). The scale is marked in mm

With the continuous Liquid Jet, a disintegration into droplets was observed in the lower pressure range, before arriving at the ocular surface at a distance of 15 mm (Figure 7).

**FIGURE 8:**
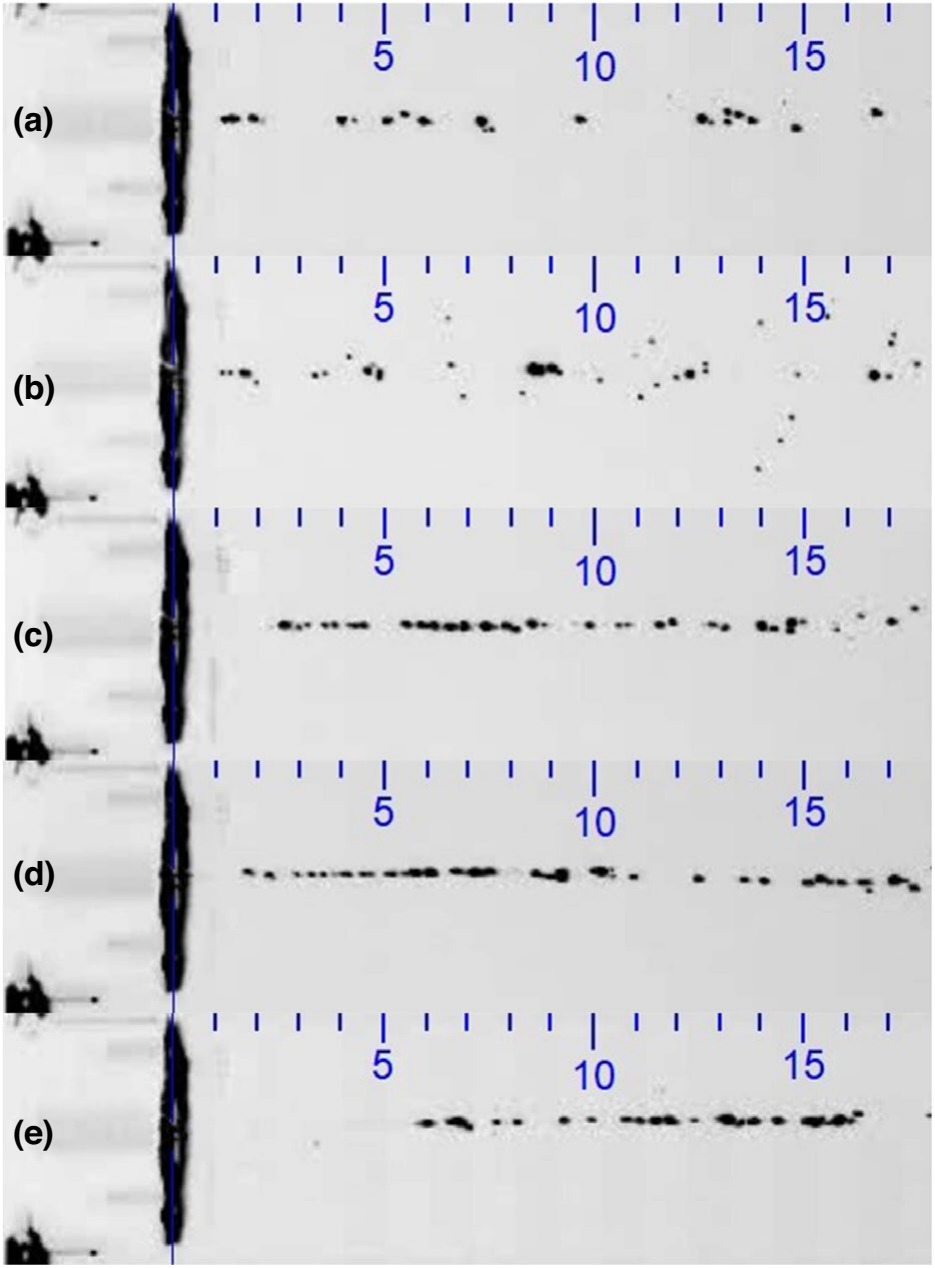
Pulsed Liquid Jet at pressures of (a) 200, (b) 300, (c) 500, (d) 800 and (e) 1000 mbar (40 intervals, 1000 µs each, with a valve opening duration of 500 µs per interval; total stimulus duration: 40 ms). The scale indicates mm and applies to both horizontal and vertical directions

As the liquid jet may be perceived differently having disintegrated into droplets compared to a solid jet in the higher pressure range, it was decided to choose a pulsed stimulus, with the aim of obtaining a Liquid Jet with similar droplet characteristics across the pressure range relevant for corneal sensitivity measurement. This could be achieved with a stimulus of 40 intervals, where the duration of each interval was 1000 µs. During each interval, the exit valve was open for 950 µs. The total stimulus duration hence remained unchanged at 40 ms (Figure 8) (Video S1).

The recording of the pulsed Liquid Jet stimulus from a semi‐lateral view showed that the stimulus arrived at a limited region on the central cornea of approximately 2 mm in diameter for a pressure of 400 mbar, representing a stimulus strength at a typical sensitivity threshold (additional film material available online).

### Stimulus mass

**FIGURE 9:**
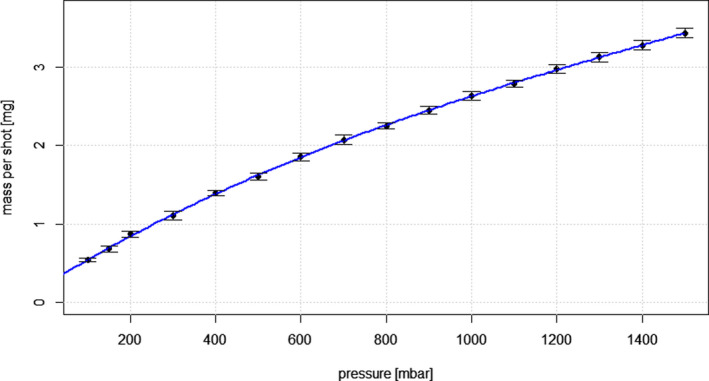
Mass per shot [mg] versus pressure [mbar] with a cubic fit. Error bars indicate the standard error means

Very little variability in mass obtained was observed during the 20 measurements at five different time points, confirming good repeatability. The zero point was found to be slightly offset, which would suggest a slight over‐estimation of the mass obtained across the tested pressure range of 100–1500 mbar (Figure 9).

The mass per shot m showed an unexpected cubic dependency on pressure p, and can be calculated by the following cubic fit:

$$m=m_{0}+m_{1}\cdot p+m_{2}\cdot p^{2}+m_{3}\cdot p^{3}$$

$$m_{0}=2.23\cdot 10^{‐1}\text{mg}$$

$$m_{1}=3.34\cdot 10^{‐03}\text{mg}\cdot {\text{mbar}}^{‐1}$$

$$m_{2}=‐1.20\cdot 10^{‐06}\text{mg}\cdot {\text{mbar}}^{‐2}$$

$$m_{3}=2.66\cdot 10^{‐10}\text{mg}\cdot {\text{mbar}}^{‐3}$$

There is little agreement with the physics of laminar liquid flows and the regression formula given must therefore be considered as empirical.

### Vertical and horizontal displacement of the Liquid Jet

Very little displacement of the Liquid Jet was observed in both horizontal and vertical directions at a distance of 15 mm from the exit nozzle (Table 1). The small values obtained display stochastic behaviour. With regard to the vertical direction, this does not allow for any physical interpretation with respect to the law of gravity.

**TABLE 1:** Vertical and horizontal displacement of the liquid jet [mm] at a distance of 15 mm from the exit nozzle

mbar	Vertical displacement [mm]	Horizontal displacement [mm]
	Mean ± SD	Mean ± SD
100	−0.04 ± 0.31	0.02 ± 0.04
300	−0.07 ± 0.26	0.03 ± 0.01
500	0.12 ± 0.21	0.03 ± 0.01
700	−0.08 ± 0.22	0.03 ± 0.01
900	0.01 ± 0.25	0.02 ± 0.02
1100	0.00 ± 0.25	0.02 ± 0.01
1300	−0.05 ± 0.23	0.08 ± 0.06
1500	−0.13 ± 0.23	0.06 ± 0.07

### Liquid Jet speed

**FIGURE 10:**
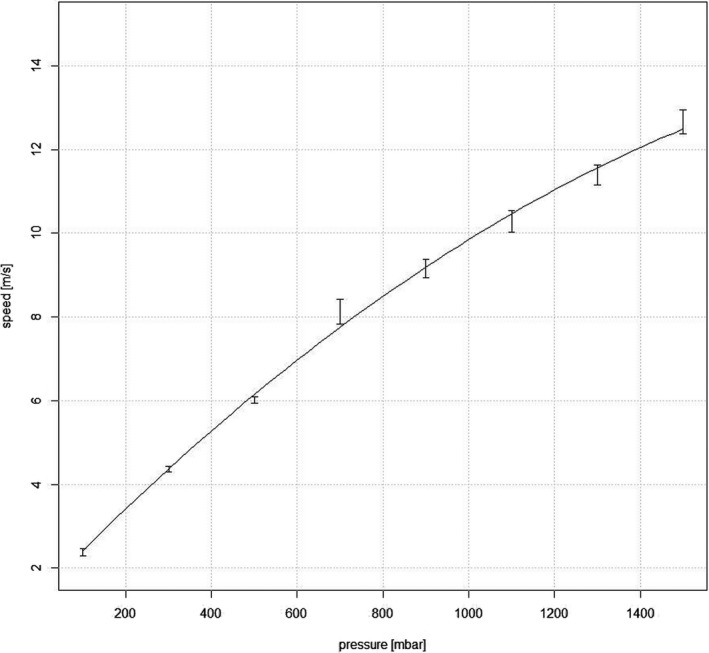
Relationship between pressure [mbar] and Liquid Jet speed [m/s]. Error bars indicate the standard error means

Up to approximately 700 mbar, an almost linear relationship was observed between pressure and Liquid Jet speed, with very little variability, obtained with the Image Shift Algorithm (Figure 10). For the pressure range of >700 mbar up to 1500 mbar, variability increased and Liquid Jet speed was found to be lower than would have been expected in a linear manner.

A quadratic fit gives a good approximation for the empirical relationship between speed *v* and pressure *p*:

$$v=v_{0}+v_{1}\cdot p+v_{2}\cdot p^{2}$$

$$v_{0}=1.377{\text{ms}}^{‐1}$$

$$v_{1}=1.06\cdot 10^{‐2}{\text{ms}}^{‐1}{\text{mbar}}^{‐1}$$

$$v_{2}=‐2.13\cdot 10^{‐6}{\text{ms}}^{‐1}{\text{mbar}}^{‐2}$$

## DISCUSSION

Non‐invasive liquid jet aesthesiometry represents a new method for the measurement of ocular surface sensitivity by utilising droplets with a specified size, shape and energy that are projected towards the ocular surface, and it has been technically validated by Ehrmann et al.^35^ With this previously presented liquid jet aesthesiometer, stimulus strength is varied with a pulse ratio method: stimulus intensity increases with the duration of the opened microvalve from a minimum of 0.15 ms to a maximum duration of typically 100 ms (total stimulus duration) that is required to elicit corneal sensation, i.e., it is quantified as the total volume or corresponding mass, ejected for one stimulus, while the pressure difference is kept constant.^35^ As mentioned above, it is unclear how this change of pulse ratio and the resulting shift in liquid volume may change stimulus strength, as a pressure difference through the exit nozzle should be necessary, either by a pressure difference or variable nozzle diameter. It is not clear how a change of pulse ratio may have an impact on stimulus strength, as the frequency of the pulsed stimulation is too high for conscious perception of individual pulses by the subject. Based on preliminary measurements on four subjects, the authors assume that threshold testing for mechanical ocular surface sensitivity is possible, as there may be differences in the way the mechanoreceptors themselves respond. It is perceivable that a change in pulse ratio may affect the physiological response of corneal nerves. However this requires neurological rather than physical explanation, and hence further research in neuroscience and neurobiology.

In order to avoid this uncertainty, it was decided to develop a modified instrument employing the same type of microvalve for the liquid jet, the SLACS, whereby stimulus intensity is varied by pressure difference with use of a digital tubing pump. Ehrmann et al. used the same exit valve as the one used in the modified instrument presented here. They showed their liquid jet aesthesiometer generated repeatable and quantifiable stimuli with regard to the critical parameters volume, velocity and lateral position of the droplets.^35^ As they limited pressure settings to 200, 250 and 300 mbar, the purpose of this study was to describe relevant physical properties of this new modified Liquid Jet aesthesiometer and validate it technically for clinical ocular surface sensitivity measurement:

The high‐speed recording of the Liquid Jet across a typical pressure range for ocular surface sensitivity measurements revealed that a continuous jet disintegrates in the low pressure range into droplets, while it remains as a continuous jet in the higher pressure range. In order to avoid a potentially different stimulus perception across the pressure range, a pulsed stimulus was created, which was shown to consist of individual droplets arriving at the ocular surface, hence creating similar stimulus characteristics across the 100–1500 mbar pressure range. The recording of the Liquid Jet arrival onto the ocular surface confirmed that it only affected a defined central region of the cornea, while the midperipheral and peripheral cornea remained unaffected.

Stimulus mass was shown to deviate from the expected linear relationship to pressure, and the null point was slightly offset. However, very little variability was observed over a range of 20 measurements per pressure, confirming good repeatability and predictability for stimulus strength. Vertical and horizontal displacement of the LJ was found to be very small across the pressure range tested; thus allowing precise presentation to the region of interest on the ocular surface.

The estimation of LJ speed obtained with the Image Shift Algorithm showed a near linear relationship for the lower pressure range up to approximately 700 mbar. For the range of >700 mbar to 1500 mbar, the obtained LJ speed was lower than would have been expected with the linear relationship, and the variability observed to be higher. This surprising result may be caused by the limitations of the method chosen to determine Liquid Jet speed: The faster the droplets move, the more difficult it becomes to identify the same droplets from one frame to another. Therefore, it is possible that the relationship between Liquid Jet speed and pressure is linear However, a more elaborate experiment would have to be carried out for confirmation of this assumption.

A fundamental question of corneal aesthesiometry concerns the physical property that is measured. Generally, in psychophysical procedures the response to a given physical stimulus is recorded. In aesthesiometry it is unclear whether the physical stimulus is pressure, force or even momentum exerted onto the cornea. The experiments conducted in this study allow for determination of force and momentum transfer applied to the cornea. However, the pressure exerted on the cornea cannot be determined because the precise surface area where the force is applied is unknown, and may vary with pressure and speed.

Liquid Jet aesthesiometry offers an interesting alternative to previously used methods for measurement of ocular surface sensitivity. Depending upon which types of nerve endings in the cornea or ocular surface are to be stimulated, then different stimulus characteristics can be chosen. For measurement of mechanical ocular surface sensation, where mechano‐nociceptors and polymodal nociceptors are excited during corneal deformation, the liquid jet temperature should match the ocular surface temperature. A cooling stimulus (at a pressure below the mechanical threshold) with variable temperatures lower than the ocular surface temperature will excite the cold temperature sensitive nerve endings. A chemical stimulus (also at a pressure below the mechanical threshold) with variable chemical composition will activate polymodal and cold receptors.

## CONCLUSIONS

This new modified Liquid Jet aesthesiometer for ocular surface sensitivity measurement (SLACS) delivered fine droplets with a pulsed stimulus mode in a repeatable manner, with precise localisation on the ocular surface. Very little variability was observed in Liquid Jet speed and mass for the typical pressure range required for clinical sensitivity measurements.

## CONFLICTS OF INTEREST

The authors report no conflicts of interest and have no proprietary interest in any of the materials mentioned in this article.

## AUTHOR CONTRIBUTION

**Daniela Sonja Nosch:** Conceptualization (equal); Formal analysis (equal); Funding acquisition (lead); Investigation (equal); Methodology (equal); Project administration (lead); Writing – original draft (lead). **Matthias Oscity:** Methodology (equal); Resources (equal); Software (equal); Validation (equal). **Peter Steigmeier:** Conceptualization (equal); Resources (equal); Software (equal); Validation (equal). **Emanuele Käser:** Data curation (equal); Investigation (equal); Methodology (equal); Resources (supporting). **Markus Loepfe:** Conceptualization (equal); Formal analysis (supporting); Investigation (supporting); Methodology (supporting); Project administration (equal); Resources (supporting); Supervision (equal). **Roland E Joos:** Conceptualization (equal); Data curation (equal); Formal analysis (equal); Funding acquisition (supporting); Investigation (equal); Methodology (equal); Software (supporting); Supervision (supporting); Validation (equal); Visualization (equal); Writing – original draft (supporting).

## Supporting information

Video S1
